# Supplementary material for: USP22 controls type III interferon signaling and SARS-CoV-2 infection through activation of STING
Source: Cell Death Dis. 2022 Aug 6;13(8):684. doi: 10.1038/s41419-022-05124-w (PMC9357023; doi:10.1038/s41419-022-05124-w)
Supplement: Supplementary file 1 — Supplementary Information [file 41419_2022_5124_MOESM1_ESM.docx]

**Supplementary Information**

**USP22 controls type III interferon signaling and SARS-CoV-2 infection through activation of STING**

*Rebekka Karlowitz, Megan L. Stanifer, Jens Roedig, Geoffroy Andrieux, Denisa Bojkova, Marco Bechtel, Sonja Smith, Lisa Kowald, Ralf Schubert, Melanie Boerries, Jindrich Cinatl Jr., Steeve Boulant, Sjoerd J. L. van Wijk ^#^*

^#^ Corresponding author: Sjoerd J. L. van Wijk, Institute for Experimental Cancer Research in Pediatrics, Goethe University Frankfurt, Komturstrasse 3a, 60528 Frankfurt am Main, Germany, Phone: +49 69 67866574, Fax: +49 69 6786659158, Email: [vanWijk@med.uni-frankfurt.de](mailto:vanWijk@med.uni-frankfurt.de), [s.wijk@kinderkrebsstiftung-frankfurt.de](mailto:s.wijk@kinderkrebsstiftung-frankfurt.de)

**Summary:** Five Supplementary Figures including Supplementary Figure legends and a Supplementary Table

**
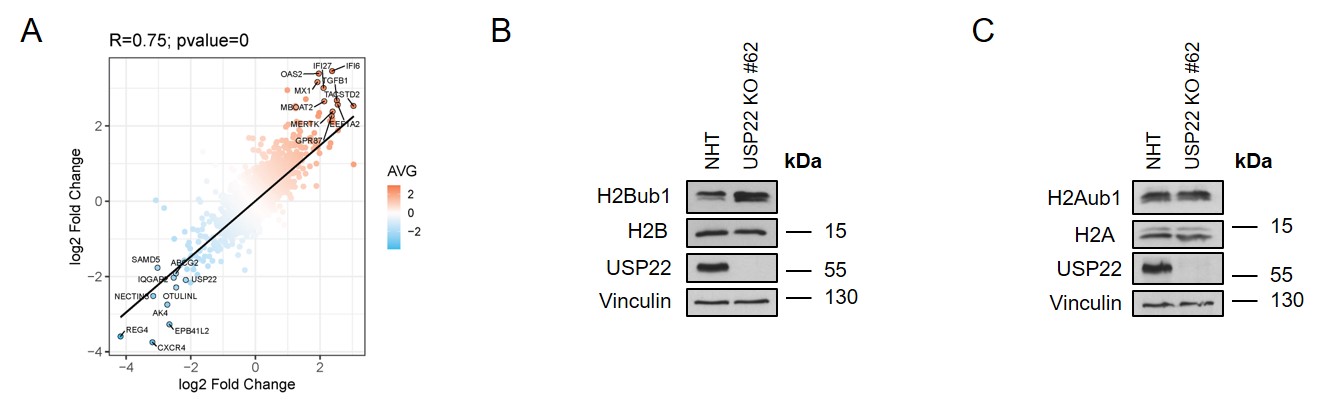
**

**Supplemental Figure 1 (related to Figure 1). A.** Scatter plot demonstrating the changes in gene expression of CRISPR/Cas9 control (NHT) HT-29 cells with two independent single-cell HT-29 USP22 KO clones (#16 and #62). Color code represents the average log2 foldchange. **B, C.** Western blot analysis of mono-ubiquitinated (H2Bub1) and total levels of Histone 2B (H2B) (**B**) and Histone 2A (H2A) (**C**) as well as USP22 in control and USP22 KO HT-29 cells (USP22 KO #62). Vinculin served as loading control. Representative blots of at least two different independent experiments are shown.


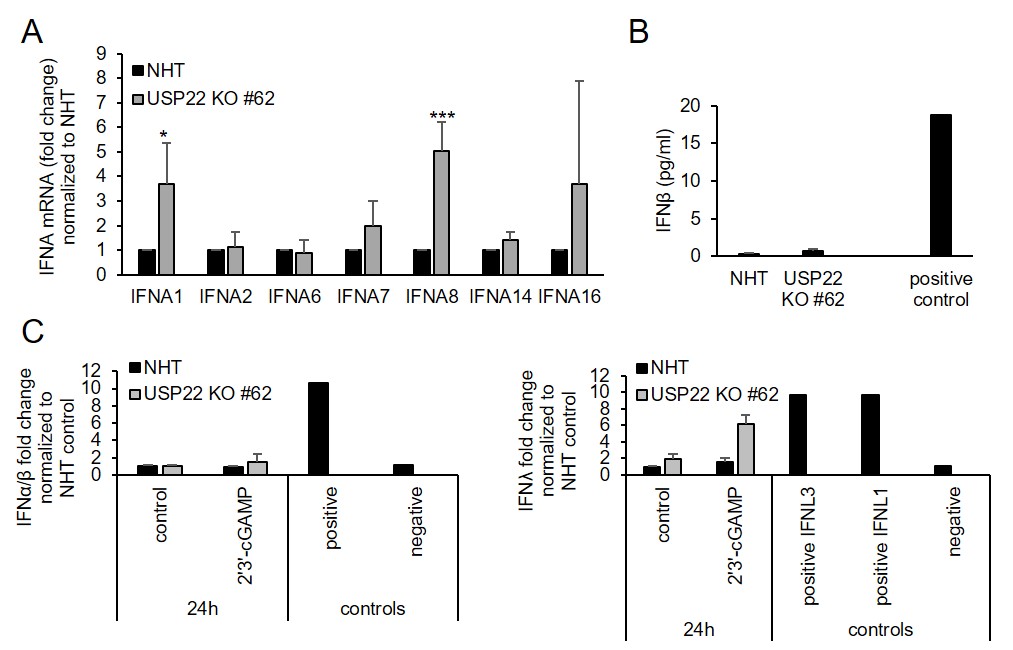


**Supplemental Figure 2 (related to Figure 3). A.** Basal mRNA expression levels of IFNA isoforms in control (NHT) and USP22 KO HT-29 cells (USP22 KO #62) using qRT-PCR. Gene expression was normalized against 28S mRNA and is presented as x-fold mRNA expression compared to NHT. Mean and SD of four independent experiments in triplicate are shown. *P < 0.05, ***P < 0.001. **B.** Basal levels of IFNβ in supernatant of control (NHT) and USP22 KO HT-29 cells (USP22 KO #62) measured by ELISA. As positive control, HT-29 USP22 KO cells were incubated for 24 hours with 10 µg/ml 2’3’-cGAMP for activation of IFN signaling. Mean and SD of three independent experiments measured in duplicate are shown. **C.** IFN-α/β (left) and IFN-λ (right) levels in supernatant of control (NHT) and USP22 KO HT-29 cells (USP22 KO #62) basal and 24 hours after stimulation with 10 µg/ml 2’3’-cGAMP, measured by luciferase assay. IFN levels were normalized to unstimulated levels in control cells. Mean and SD of three independent experiments are shown.

**
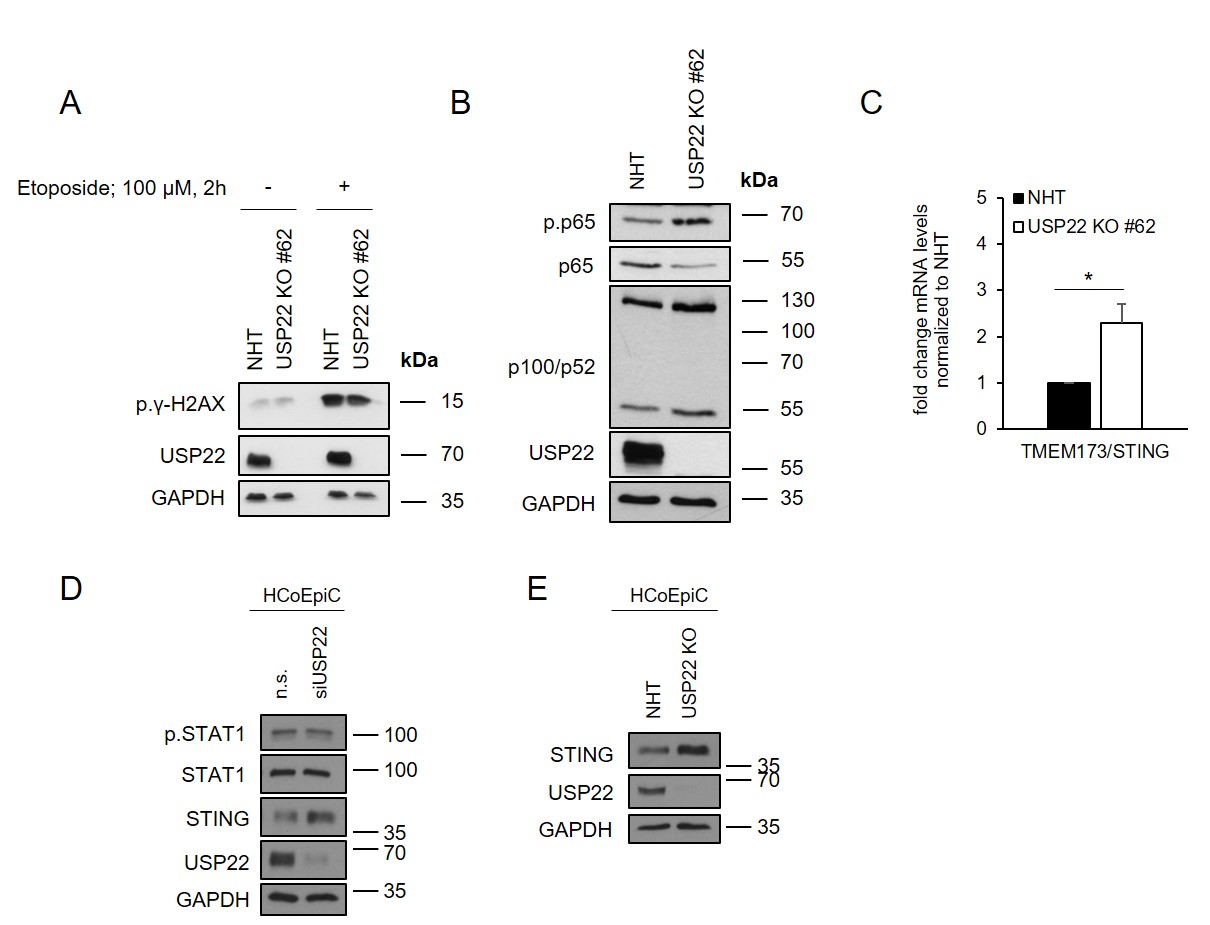
**

**Supplemental Figure 3 (related to Figure 4). A.** Western blot analysis of phosphorylated γ-H2AX (p.γ-H2AX) and USP22 expression levels in control (non-human target: NHT) and CRISPR/Cas9-generated USP22 knock-out (KO) HT-29 cells (USP22 KO) subjected to vehicle or etoposide (100 µM) for 2 h. GAPDH served as loading control. Representative blots of at least two different independent experiments are shown. **B.** Western blot analysis of phosphorylated and total p65, p100/p52 and USP22 expression levels in control and USP22 KO HT-29 cells (USP22 KO #62). GAPDH served as loading control. Representative blots of at least two different independent experiments are shown. **C.** Basal mRNA expression levels of TMEM173/STING in control and USP22 KO HT-29 cells (USP22 KO #62) using qRT-PCR. Gene expression was normalized against 28S mRNA and is presented as x-fold mRNA expression compared to NHT. Mean and SD of three independent experiments in triplicate are shown. *P < 0.05. **D.** Western blot analysis of phosphorylated and total STAT1, total STING and USP22 expression levels in human epithelial HCoEpiC cells that were subjected to RNAi mediated silencing of USP22 and a non-silencing (n.s.) siRNA control for 96 hours. GAPDH served as loading control. Representative blots of three different independent experiments are shown. **E.** Western blot analysis of STING and USP22 expression levels in CRISPR/Cas9-generated control (NHT) and USP22 knock-out (KO) HCoEpiC cells. GAPDH served as loading control. Representative blots of two independent experiments are shown.


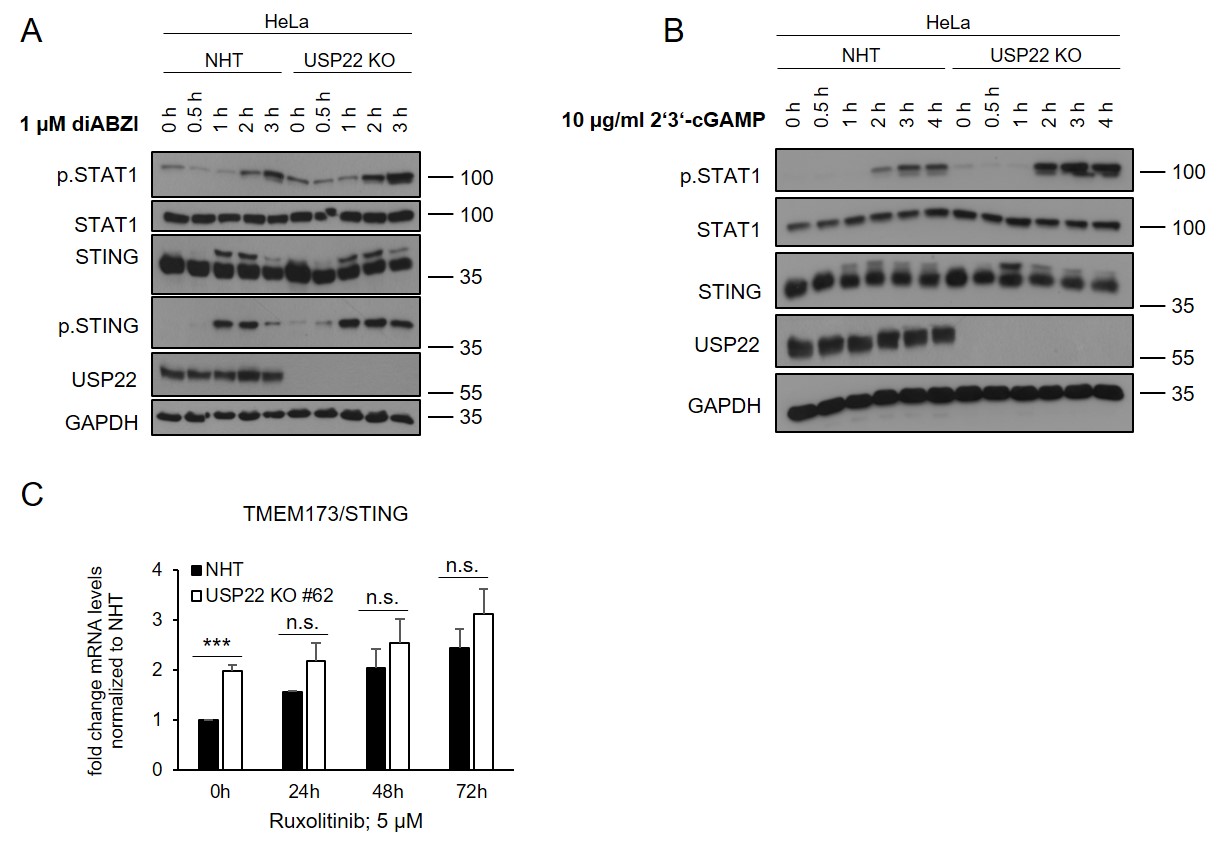
**Supplemental Figure 4 (related to Figure 5). A.** Western blot analysis of phosphorylated and total STAT1, phosphorylated and total STING, and USP22 expression levels in HeLa cells treated with 1 µM diABZI for indicated time points. GAPDH served as loading control. Representative blots of two different independent experiments are shown. **B.** Western blot analysis of phosphorylated and total STAT1, STING, and USP22 expression levels in HeLa cells treated with 10 µg/ml 2’3’-cGAMP for indicated time points. GAPDH served as loading control. Representative blots of two different independent experiments are shown. **C.** mRNA expression levels of TMEM173/STING in control (non-human target: NHT) and CRISPR/Cas9-generated USP22 knock-out (KO) HT-29 cells (USP22 KO) using qRT-PCR. Cells were treated with ruxolitinib (5 µM) for the indicated timepoints. Gene expression was normalized against 28S mRNA and is presented as x-fold mRNA expression compared to NHT. Mean and SD of three independent experiments in triplicate are shown. ***P < 0.001, n.s. not significant.

**
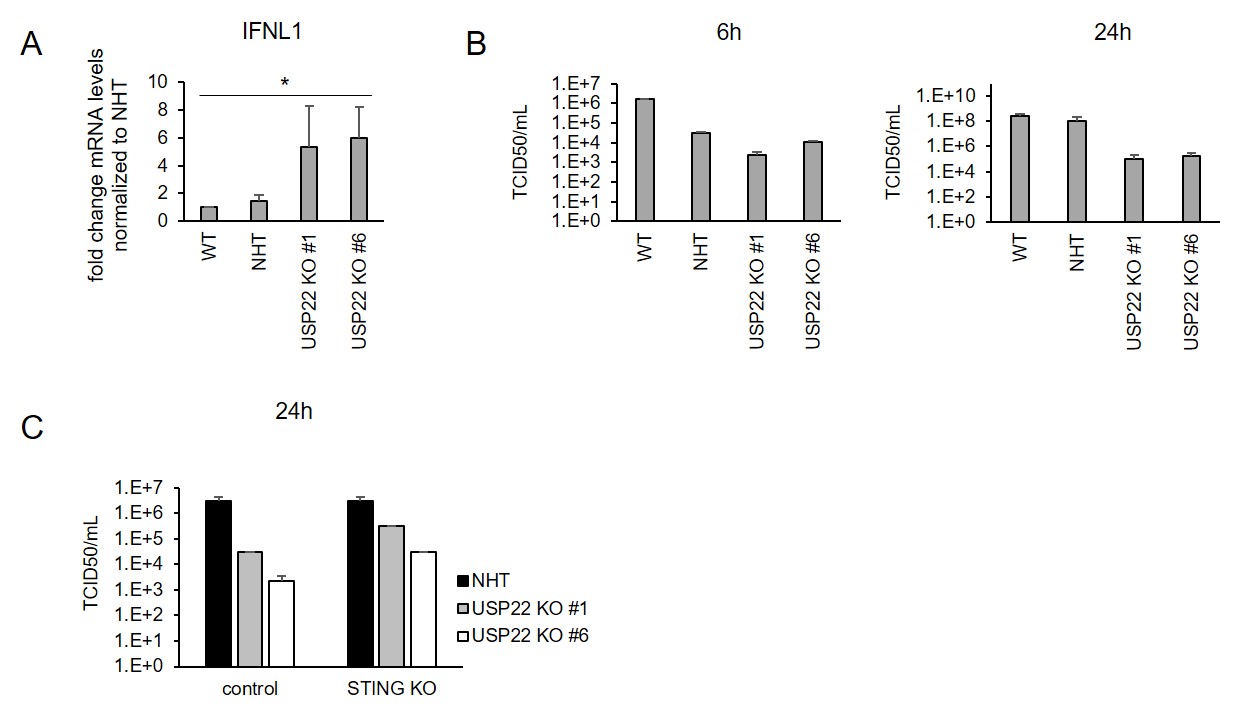
Supplemental Figure 5 (related to Figure 6). A.** Basal mRNA expression levels of IFNL1 in wild-type (WT), control (non-human target: NHT) and CRISPR/Cas9-generated USP22 knock-out (KO) Caco-2 single clones (USP22 KO #1 and #6). Gene expression was normalized against 28S mRNA and is presented as x-fold mRNA expression compared to NHT. Mean and SD of four (IFNA, IFNB) or three (INFL1) independent experiments in triplicate are shown. *P < 0.05. **B.** TCID50/mL, determined via titration of supernatant from SARS-CoV-2-infected WT, control and USP22 KO Caco-2 cells (USP22 KO #1 and #6) 6 and 24 hpi on Vero cells. Mean and SD of three independent experiments in triplicate are shown. **C.** Idem as ***B.***, 24 hpi, additional supernatants tested from NHT- and USP22 KO #1- or #6-STING dKO Caco-2 cells.

**Supplementary Table 1: List of qRT-PCR primers used in this study**

|  | *Forward primer* | *Reverse primer* |
| --- | --- | --- |
|  |  |  |
| AK4 | CACTGGTGAACCGTTAGTCCA | AGCACTCCTCGGCTCTTGT |
| BST2 | CCACCTGCAACCACACTG | CCTGAAGCTTATGGTTTAATGTAGTG |
| COV1 | GCCTCTTCTGTTCCTCATCAC | AGACAGCATCACCGCCATTG |
| CXCR4 | GGCCCTCAAGACCACAGTCA | TTAGCTGGAGTGAAAACTTGAAG |
| DDX58 | TGTGGGCAATGTCATCAAAA | GAAGCACTTGCTACCTCTTGC |
| DDX60 | AATCCCACAGGACTGCACA | TCGACCAAATACCTTCTGCAA |
| IFI27 | GTGGCCAAAGTGGTCAGG | CCAATCACAACTGTAGCAATCC |
| IFI6 | AACCGTTTACTCGCTGCTGT | GGGCTCCGTCACTAGACCT |
| IFIH1 | TTTTGCAGATTCTTCTGTAGTTTCA | TGCTGTTATGTCCAAGACTTTCA |
| IFIT1 | CTTGTGGGTAATACAGTGGAGATG | GCTCCAGACTATCCTTGACCTG |
| IFNA1 | TCAAAGACTCTCACCCCTGC | CAGTGTAAAGGTGCACATGACG |
| IFNA10 | CACGACGCGTTGAATCAAAAT | ACATTAACCACAATGTAAAGCGAC |
| IFNA14 | CATCTTCGGGATTCCCAATGGC | TTACAGCCCAGAGAGCAGCTT |
| IFNA16 | GGATTCATCTGCTGCTTGGGATG | GAGTCCTCATTCATCAGGGCAA |
| IFNA17 | TGCTGGTGCTCAGCTACAAA | TCCTCCTGGGGAAGTCCAAA |
| IFNA2 | TTTCAACCAGTCTAGCAGCATCT | TCAAGGTCCTCCTGCTACCC |
| IFNA21 | TCCACACTTCTATGACTTCTGCC | TGCCTGCACAGGTAAACATGA |
| IFNA4 | AGAGGCCGAAGTTCAAGGTTA | ACTGGTGGCCATCAAACTCC |
| IFNA5 | CAAGGTTCAGGGTCACTCAAT | CACCAGGGCCATCAGTAAAAC |
| IFNA6 | ATCTGTTGCTTGGGATGAGAGG | AGGCACAAGGGCTGTACTTTT |
| IFNA7 | CCCACCTCAGGTAGCCTAGTGAT | TCACAGCCCAGAGAGCAGAT |
| IFNA8 | CTGTTCAGCTGTATGGGCAC | GCACAATCAGGGTTGGAGTTC |
| IFNB1 | ATGACCAACAAGTGTCTCCTCC | GGAATCCAAGCAAGTTGTAGCTC |
| IFNL1 | GGACGCCTTGGAAGAGTCAC | AGCTGGGAGAGGATGTGGT |
| IRF9 | AGCCTGGACAGCAACTCAG | GAAACTGCCCACTCTCCACT |
| ISG15 | GAGGCAGCGAACTCATCTTT | AGCATCTTCACCGTCAGGTC |
| OAS2 | TGCAGGGAGTGGCCATAG | TCTGATCCTGGAATTGTTTTAAGTC |
| OAS3 | TCCCATCAAAGTGATCAAGGT | ACGAGGTCGGCATCTGAG |
| panIFNA | TCCATGAGVTGATBCAGCAGA | ATTTCTGCTCTGACAACCTCCC |
| PARP9 | CTGTCTGCACCGAGGAGAG | GCGCTTCAAAGCATAGACTGT |
| SLFN5 | AGCAAGCCTGTGTGCATTC | ACCACTCTGTCTGAAAATACTGGA |
| TBP | CCACTCACAGACTCTCACAAC | CCACTCACAGACTCTCACAAC |
| TGFB1 | ACTACTACGCCAAGGAGGTCAC | TGCTTGAACTTGTCATAGATTTCG |
| TGM2 | GGCACCAAGTACCTGCTCA | AGAGGATGCAAAGAGGAACG |
| TMEM173 | ACATTCGCTTCCTGGATAAACT | CTGCTGTCATCTGCAGGTTC |
| USP18 | TCCCGACGTGGAACTCAG | CAGGCACGATGGAATCTCTC |
| USP22 | GAAGATCACCACGTATGTGTCC | CATTCATCCTGCTCTCTTTGC |
