# Supplementary material for: USP22 controls type III interferon signaling and SARS-CoV-2 infection through activation of STING
Source: Cell Death Dis. 2022 Aug 6;13(8):684. doi: 10.1038/s41419-022-05124-w (PMC9357023; doi:10.1038/s41419-022-05124-w)

Figure 2D, membrane #1

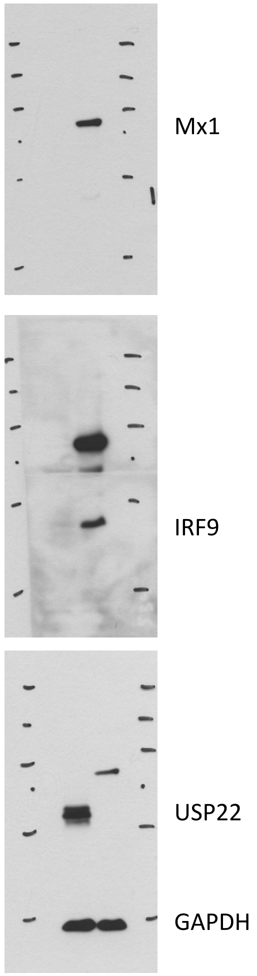

Figure 2D, membrane #2

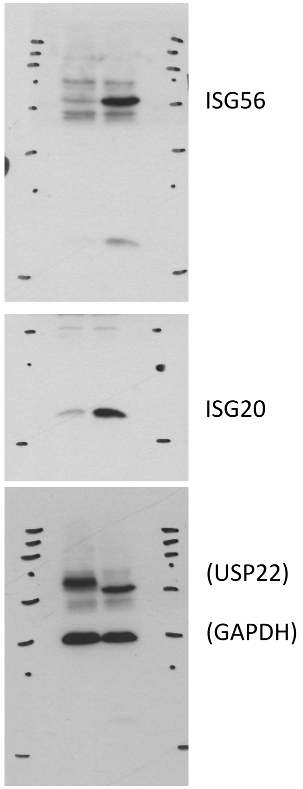

Figure 3B, membrane #1

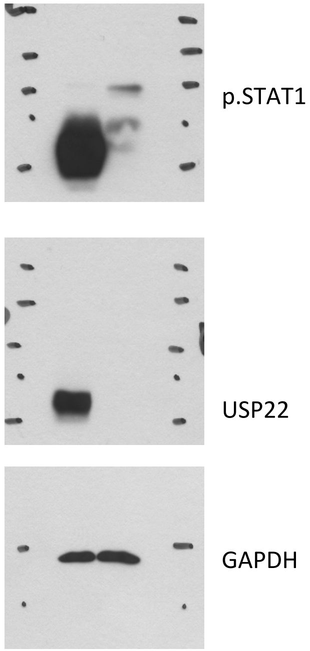

Figure 3B, membrane #2

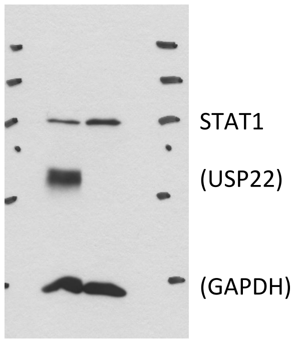

Figure 3C, membrane #1

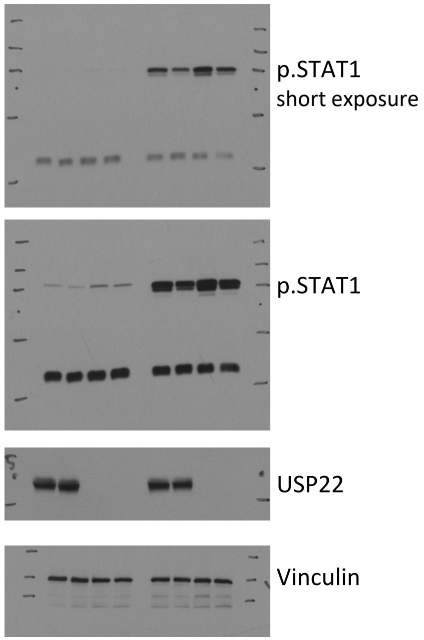

Figure 3C, membrane #2

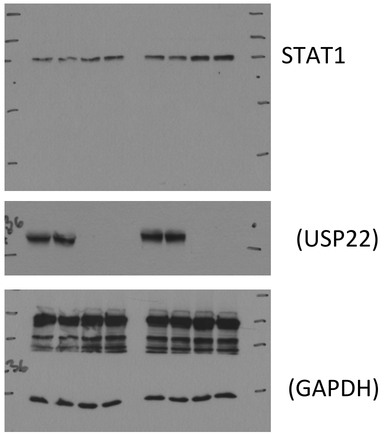

Figure 4A, membrane #1: MDA5 (first two lanes)

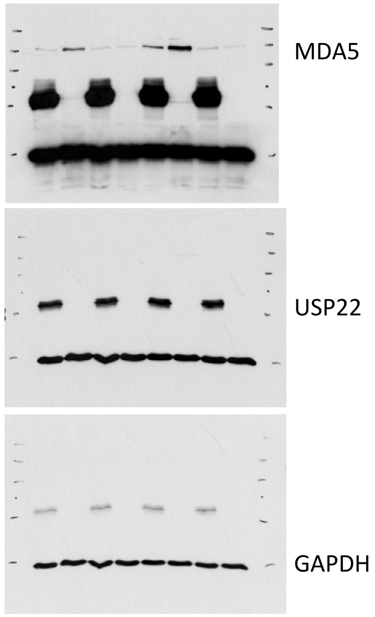

Figure 4A, membrane #3: TLR3

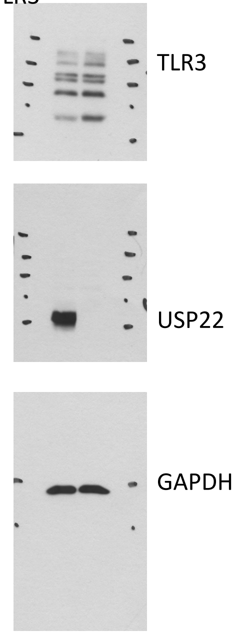

Figure 4A, membrane #2: RIG-I (first two lanes)

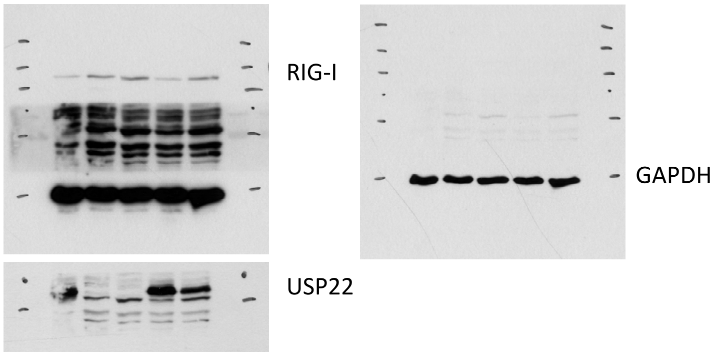

Figure 4B, membrane #1

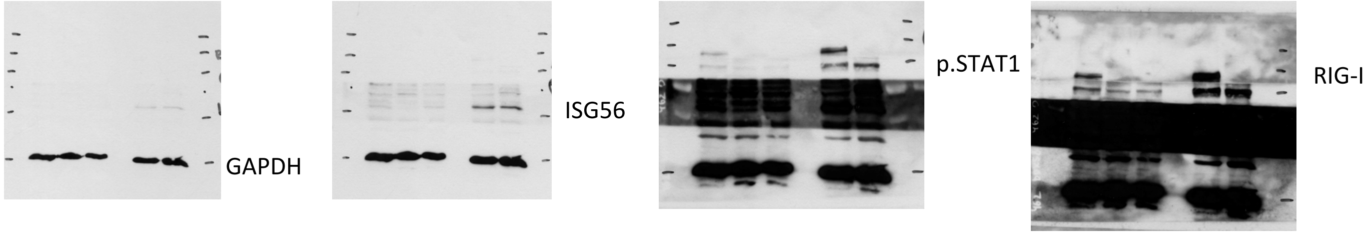

Figure 4B, membrane #2

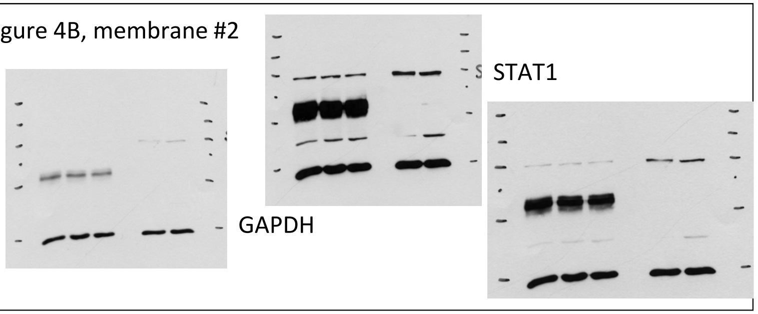

Figure 4C, membrane #1

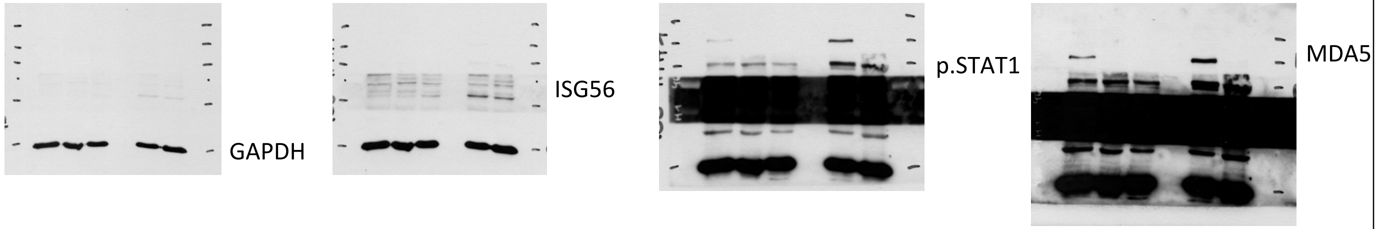

Figure 4C, membrane #2

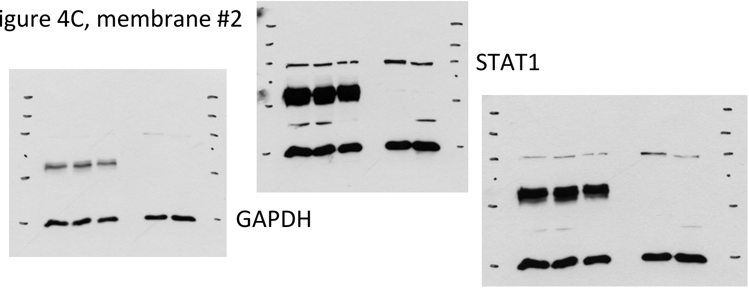

Figure 4D, membrane #1

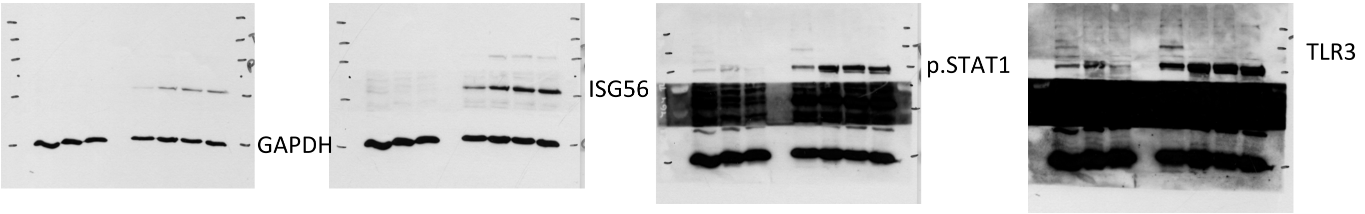

Figure 4D, membrane #2

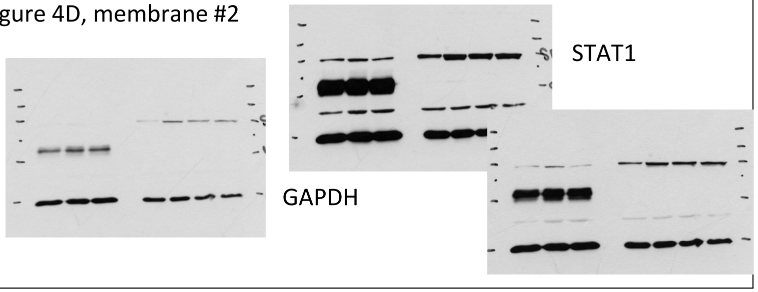

Figure 4B, membrane #1

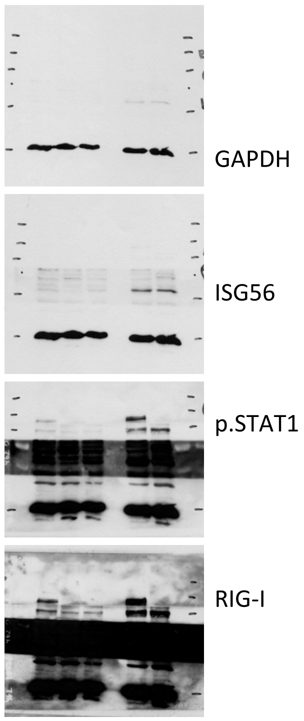

Figure 4B, membrane #2

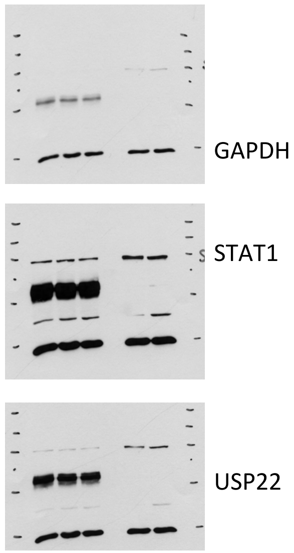

Figure 4C, membrane #1

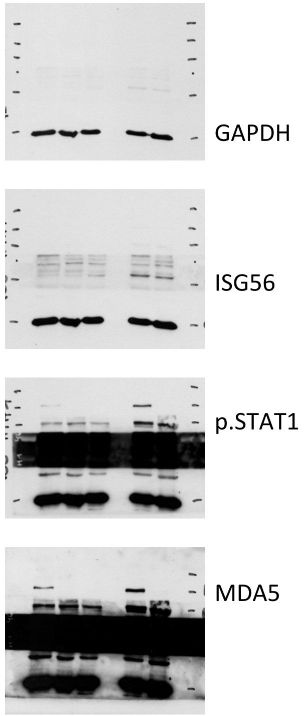

Figure 4C, membrane #2

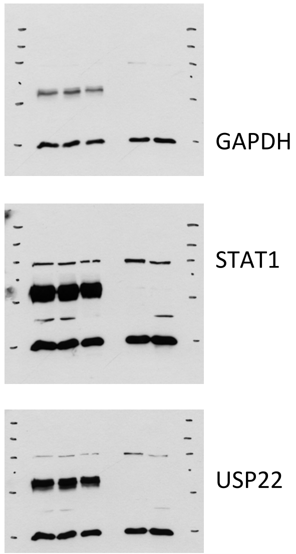

Figure 4D, membrane #1

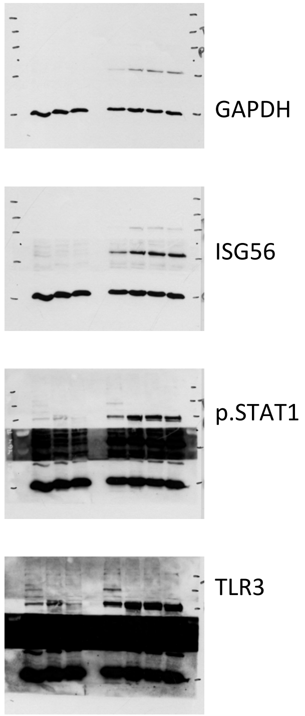

Figure 4D, membrane #2

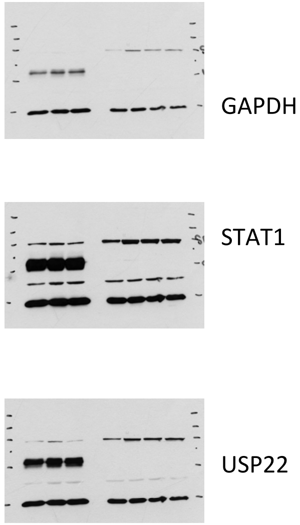

Figure 4E, membrane #1

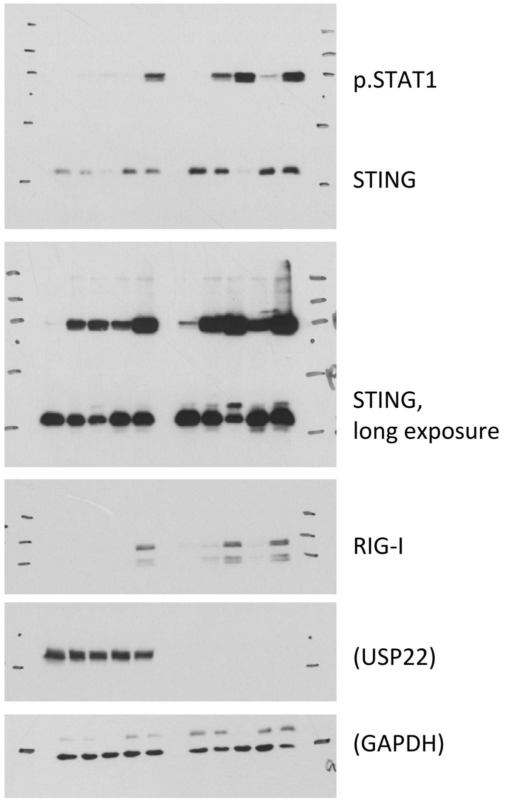

Figure 4E, membrane #2

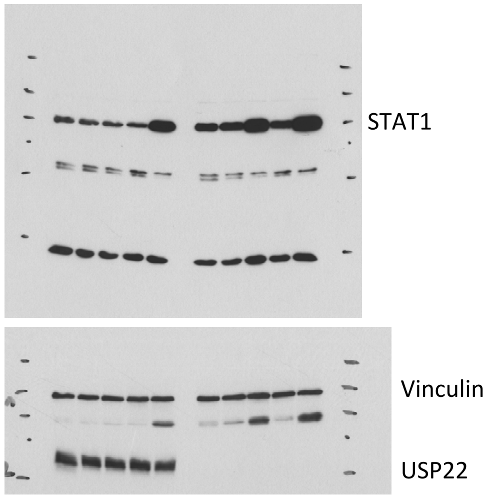

Figure 4G, membrane #1

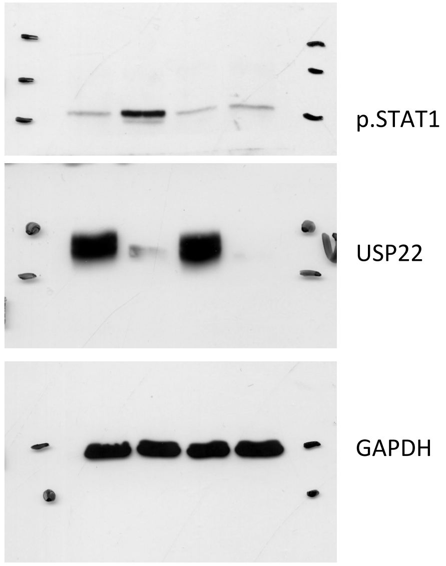

Figure 4G, membrane #2

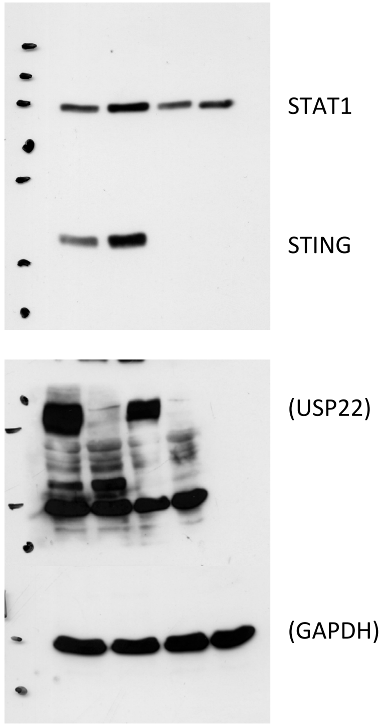

Figure 5A, membrane #1

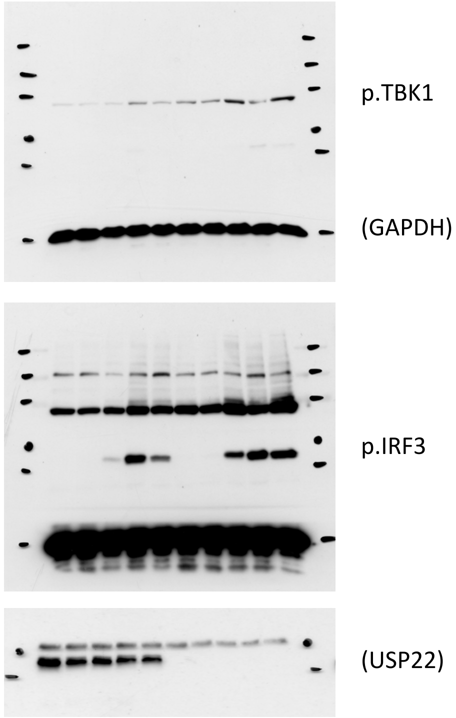

Figure 5A, membrane #2

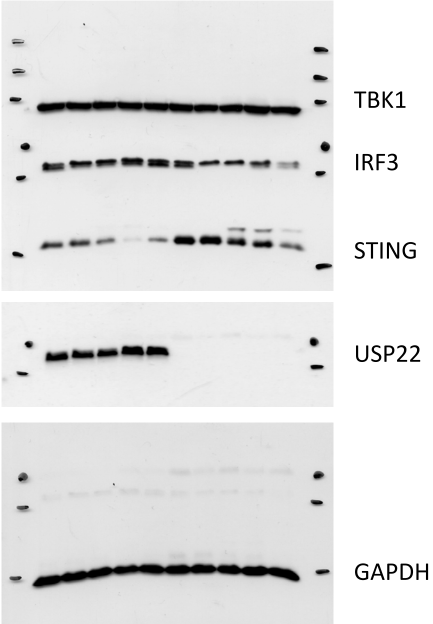

Figure 5C, membrane #1

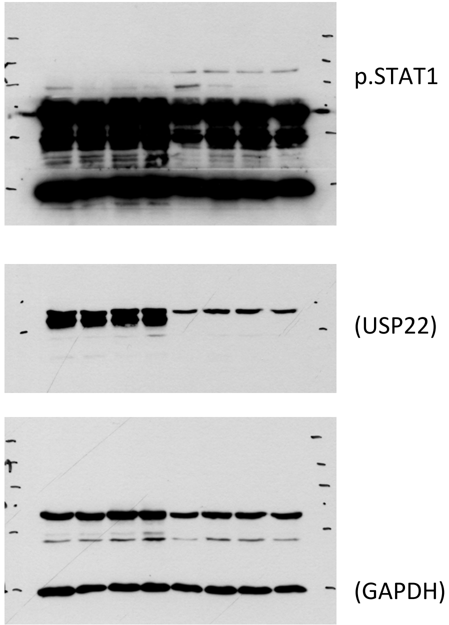

Figure 5C, membrane #2

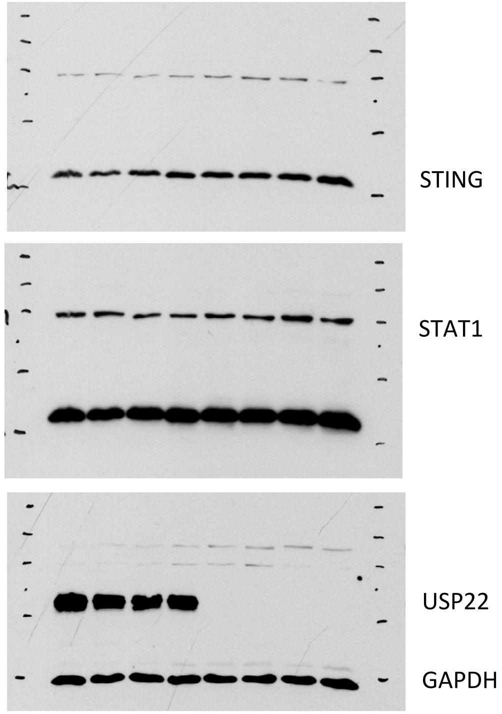

Figure 5D, membrane #1

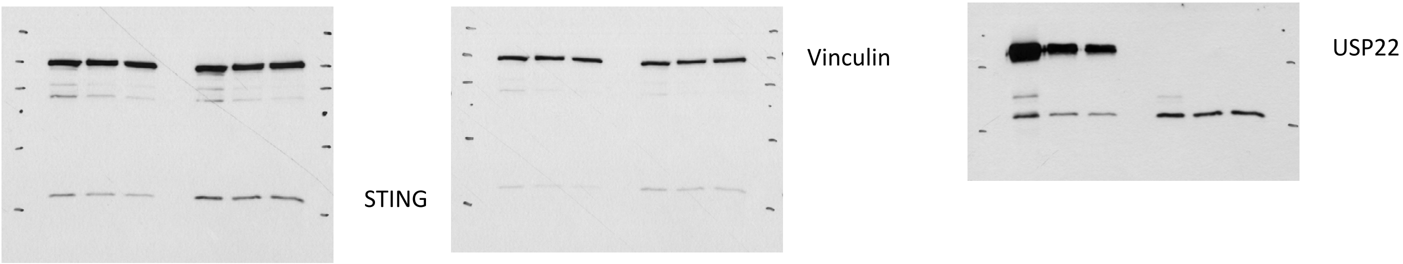

Figure 5E, membrane #1

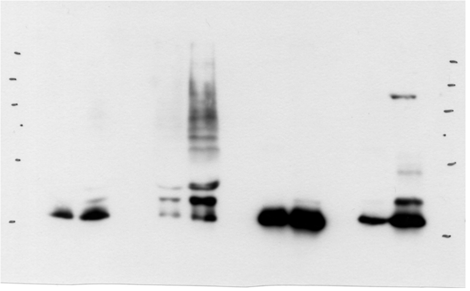

STING

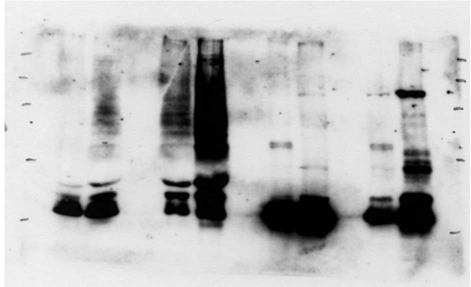

STING,
long exposure

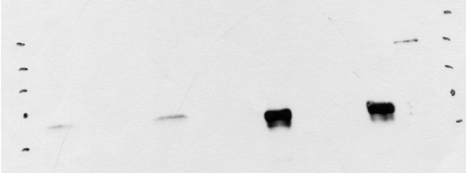

USP22

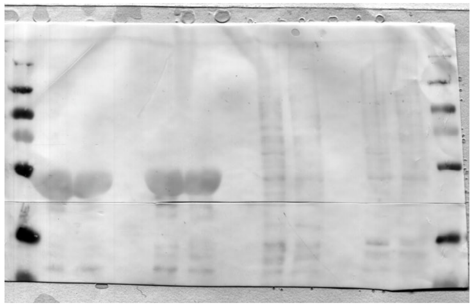

Ponceau staining for GST

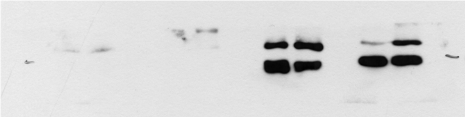

GAPDH

Figure 6A, membrane #1 (lanes 1-4)

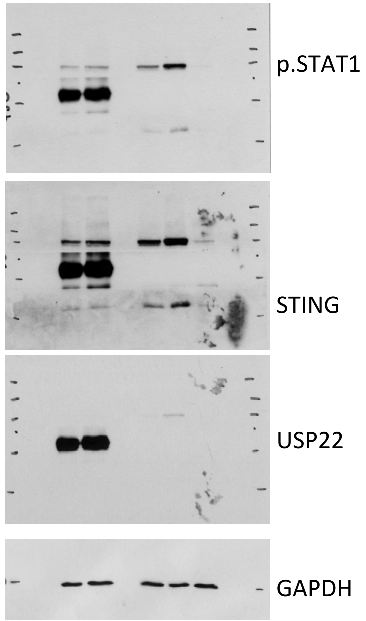

Figure 6A, membrane #2 (lanes 1-4)

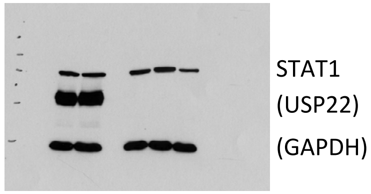

Figure 6C, membrane #1

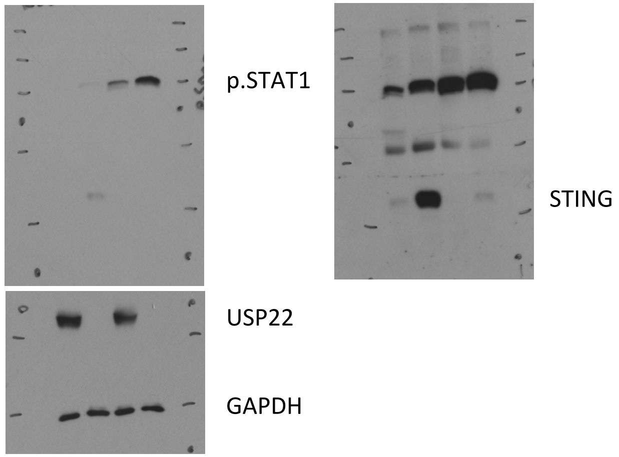

Figure 6C, membrane #2

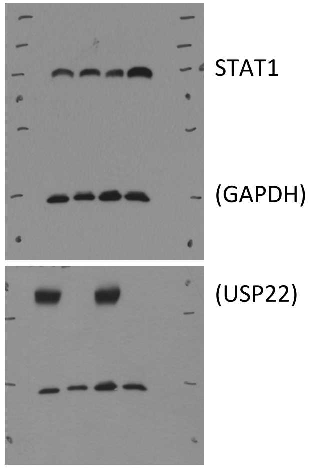

Figure 6G, membrane #1 (lanes 2-7)

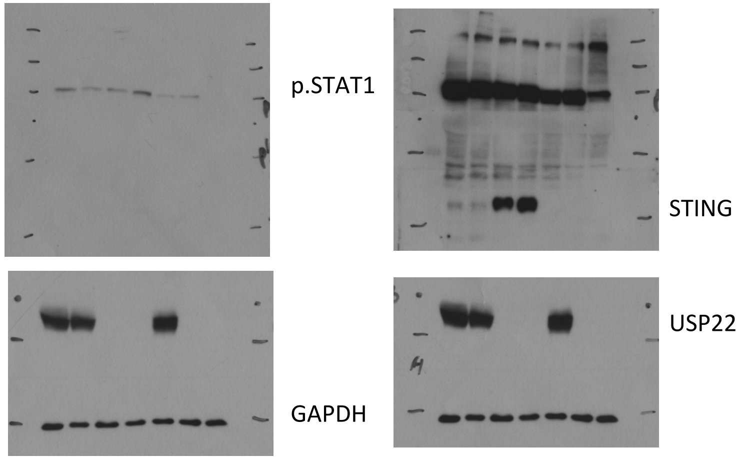

Figure 6G, membrane #2 (lanes 2-7)

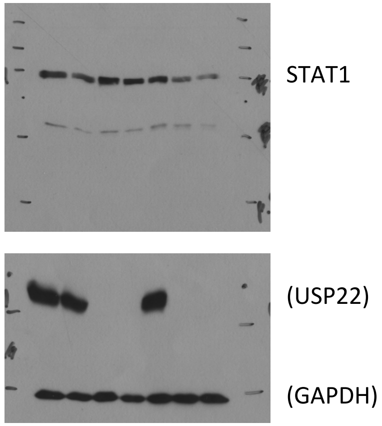

Supplemental Figure 1B, membrane #1

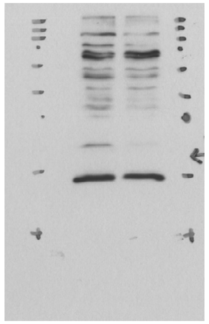
H2B

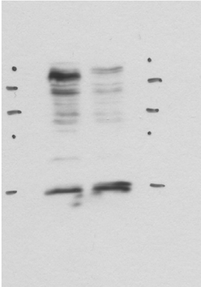
(USP22)

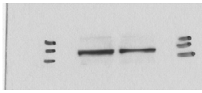
(Vinculin)

Supplemental Figure 1B, membrane #2

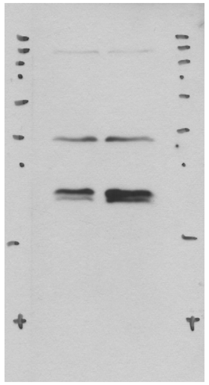
H2Bub1

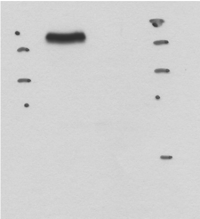
USP22

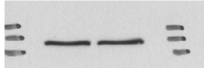
Vinculin

Supplemental Figure 1C, membrane #1

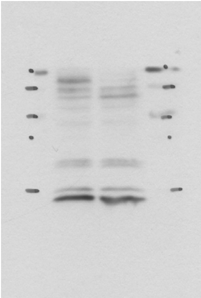
H2A

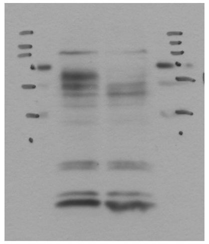
(USP22)

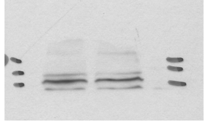
(Vinculin)

Supplemental Figure 1C, membrane #2

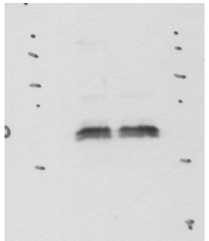
H2Aub1

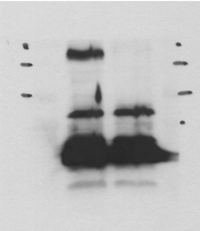
USP22

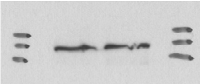
Vinculin

Supplemental Figure 3A, membrane #1

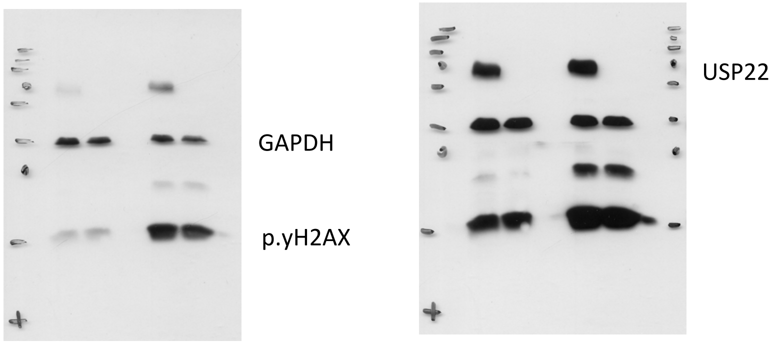

Supplemental Figure 3B, membrane #1

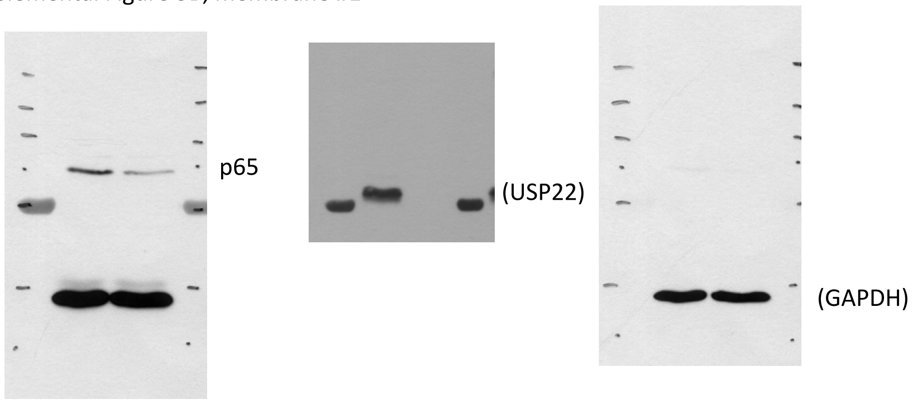

Supplemental Figure 3B, membrane #2

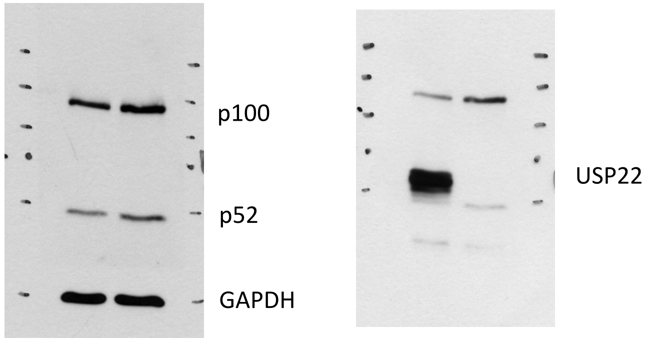

Supplemental Figure 3B, membrane #3

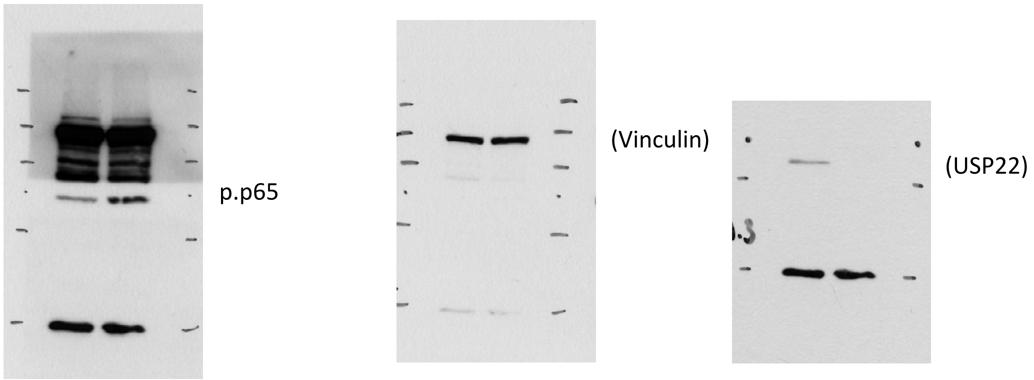

Supplemental Figure 3D, membrane #1 (lanes 2-3)

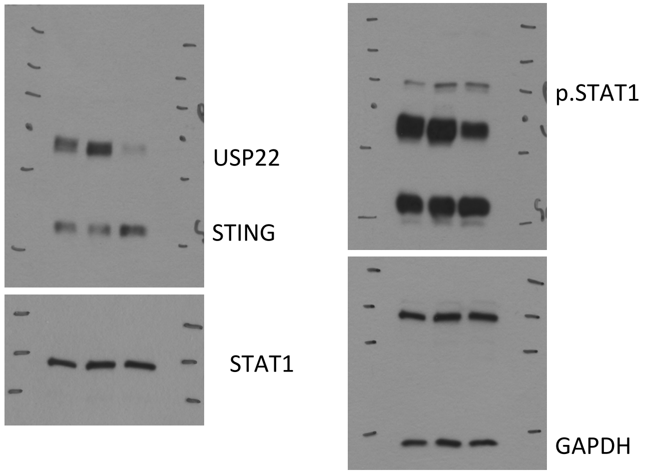

Supplemental Figure 3E, membrane #1

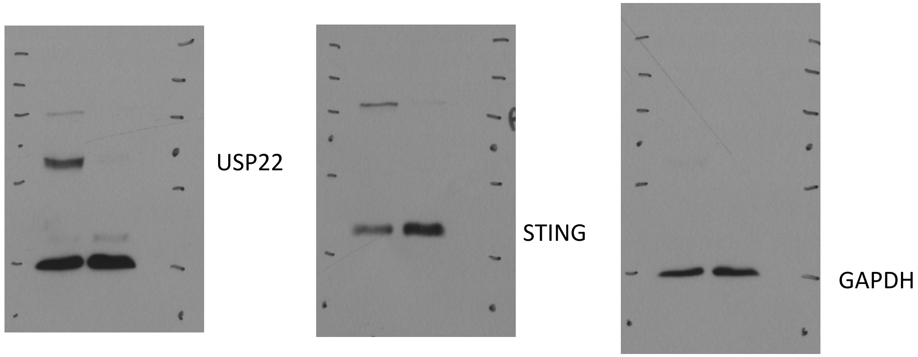

Supplemental Figure 4A, membrane #1

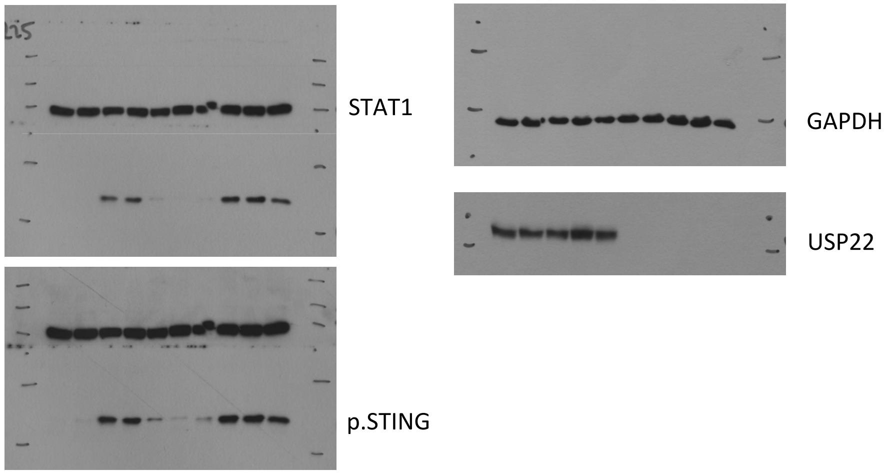

Supplemental Figure 4A, membrane #2

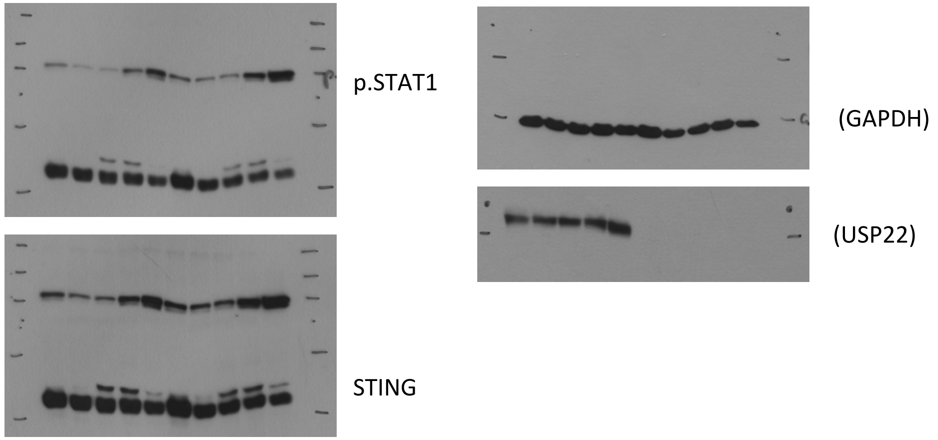

Supplemental Figure 4B, membrane #1

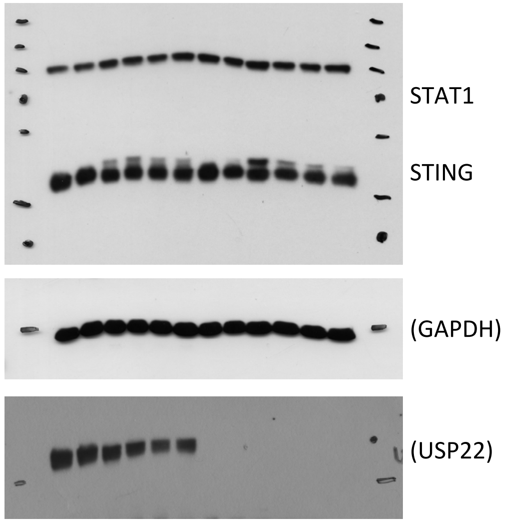

Supplemental Figure 4B, membrane #2

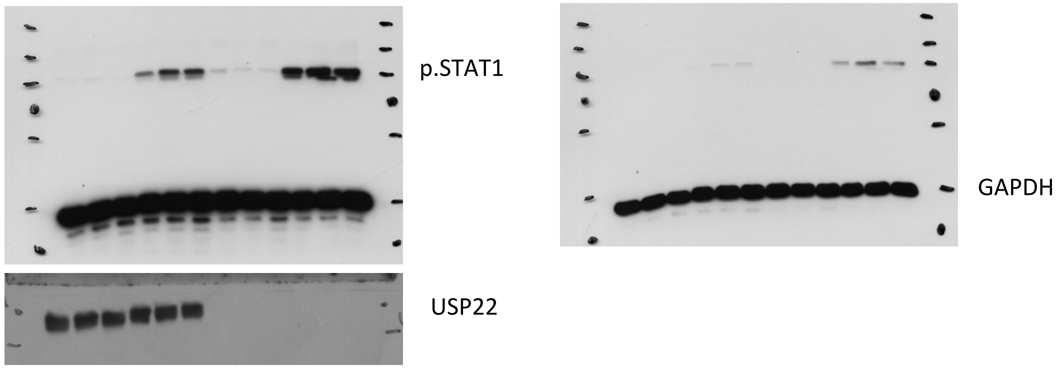

Supplement: Supplementary file 2 — Uncropped Western blots [file 41419_2022_5124_MOESM2_ESM.pdf]
