# Supplementary material for: USP22 controls type III interferon signaling and SARS-CoV-2 infection through activation of STING
Source: Cell Death Dis. 2022 Aug 6;13(8):684. doi: 10.1038/s41419-022-05124-w (PMC9357023; doi:10.1038/s41419-022-05124-w)
Supplement: Supplementary file 4 — Change of authorship form [file 41419_2022_5124_MOESM4_ESM.pdf]

SPRINGER NATURE

Change of authorship request form - Journals (pre-acceptance)

Important information. Please read.

- This form should be used by authors to request any change in authorship (adding/deleting authors) including changes in corresponding authors. This form should not be used for name changes. Please fully complete all sections. Use black ink and block capitals and provide each author's full name with the given name first followed by the family name.
- By signing this declaration, all authors guarantee that the order of the authors are in accordance with their scientific contribution, if applicable as different conventions apply per discipline, and that only authors have been added who made a meaningful contribution to the work.
- Please note, in author collaborations where there is formal agreement for representing the collaboration, it is sufficient for the representative or legal guarantor (usually the corresponding author) to complete and sign the Authorship Change Form on behalf of all authors, **next to the added/removed author(s). (Complete Section 3, followed by Section 6.)**
  In author collaborations where there is no formal agreement for representing the collaboration and **there are more than 10 authors**, one may sign for all, provided the signer appends correspondence that attests that each of the authors have agreed to the change **and the added/removed authors sign the form. (Complete Section 3, followed by Section 6.)**
- Please note, we cannot investigate or mediate any authorship disputes. If you are unable to obtain agreement from all authors (including those who you wish to be removed) you must refer the matter to your institution(s) for investigation. Please inform us if you need to do this.
- If you are not able to return a fully completed form within **30 days** of the date that it was sent to the author requesting the change, we may have to withdraw your manuscript. We cannot publish manuscripts where authorship has not been agreed by all authors (including those who have been removed).
- Incomplete forms will be rejected.
- Please return/upload this form, fully completed, to the Journals Editorial Office. The Journal and/or Publisher will consider the information you have provided to decide whether to approve the proposed change in authorship. We may decide to contact your institution for more information or undertake a further investigation, if appropriate, before making a final decision.

SPRINGER NATURE

Change of authorship request form - Journals (pre-acceptance)

Section 1: Please provide the current title of manuscript

Manuscript ID no.: CDDIS-22-0894R

Title: USP22 controls type III interferon signaling and SARS-CoV-2 infection through activation of STING

Section 2: Please provide the previous authorship, in the order shown on the manuscript before the changes were introduced. Please indicate the corresponding author by adding (CA) behind the name.

	First name(s)	Family name	ORCID or SCOPUS id, if available
1st author	Rebekka	Karlowitz	0000-0001-5290-9468
2nd author	Megan L.	Stanifer	0000-0001-6532-7651
3rd author	Jens	Roedig	
4th author	Geoffroy	Andrieux	
5th author	Denisa	Bojkova	
6th author	Sonja	Smith	
7th author	Lisa	Kowald	
8th author	Ralf	Schubert	
9th author	Melanie	Boerries	
10th author	Jindrich	Cinatl Jr.	

Please use an additional sheet if there are more than 10 authors.

SPRINGER NATURE Springer Nature is one of the world's leading global research, educational and professional publishers, created in May 2015 through the combination of Nature Publishing Group, Palgrave Macmillan, Macmillan Education and Springer Science+Business Media.

SPRINGER NATURE

Change of authorship request form - Journals (pre-acceptance)

Section 1: Please provide the current title of manuscript

Manuscript ID no.: CDDIS - 22 - 0894R

Title: USP22 controls type III interferon signaling and SARS-CoV-2 infection through activation of STING

Section 2: Please provide the previous authorship, in the order shown on the manuscript before the changes were introduced. Please indicate the corresponding author by adding (CA) behind the name.

	First name(s)	Family name	ORCID or SCOPUS id, if available
1 1st author	Steeve	Boulant	
1 2nd author	Sjoerd J.L.	van Wijk (CA)	
3rd author			
4th author			
5th author			
6th author			
7th author			
8th author			
9th author			
10th author			

Please use an additional sheet if there are more than 10 authors.

SPRINGER NATURE Springer Nature is one of the world's leading global research, educational and professional publishers, created in May 2015 through the combination of Nature Publishing Group, Palgrave Macmillan, Macmillan Education and Springer Science+Business Media.

SPRINGER NATURE

Change of authorship request form - Journals (pre-acceptance)

Section 3: Please provide a justification for change. Please use this section to explain your reasons for changing the authorship of your manuscript, e.g. what necessitated the change in authorship? Please refer to the (journal) policy pages for more information about authorship. Please explain why omitted authors were not originally included and/or why authors were removed on the submitted manuscript.

Marco Bechtel provided substantial experimental help and expertise with HCoEpiC cells, that were used in the revision process.

Section 4: Proposed new authorship. Please provide your new authorship list in the order you would like it to appear on the manuscript. Please indicate the corresponding author by adding (CA) behind the name. If the Corresponding Author has changed, please indicate the reason under section 3.

	First name(s)	Family name (this name will appear in full on the final publication and will be searchable in various abstract and indexing databases)	Affiliated institute	E-mail address
1st author	Rebekka	Karlowitz	Institute for Experimental Cancer Research in Pediatrics, Goethe University Frankfurt	r.karlowitz@kinderkrebsstiftung-frankfurt.de
2nd author	Megan L.	Stanifer	Department of Infectious Diseases/Molecular Virology, Medical Faculty, Center for Integrative Infectious Diseases Research (CIID), University of Heidelberg<br>Department of Molecular Genetics and Microbiology, University of Florida College of Medicine, Gainesville, Florida	m.stanifer@ufl.edu
3rd author	Jens	Roedig	Institute for Experimental Cancer Research in Pediatrics, Goethe University Frankfurt	jens.roedig@gmx.net
4th author	Geoffroy	Andrieux	Institute of Medical Bioinformatics and Systems Medicine, Medical Center-University of Freiburg, Faculty of Medicine, University of Freiburg	geoffroy.andrieux@uniklinik-freiburg.de
5th author	Denisa	Bojkova	Institute of Medical Virology, University Hospital Frankfurt, Goethe University	Denisa.Bojkova@kgu.de
6th author	Marco	Bechtel	Institute of Medical Virology, University Hospital Frankfurt, Goethe University	Marco.Bechtel@kgu.de
7th author	Sonja	Smith	Institute for Experimental Cancer Research in Pediatrics, Goethe University Frankfurt	s.smith@kinderkrebsstiftung-frankfurt.de
8th author	Lisa	Kowald	Institute for Experimental Cancer Research in Pediatrics, Goethe University Frankfurt	Lisa.Kowald@gmx.de
9th author	Ralf	Schubert	Division for Allergy, Pneumology and Cystic Fibrosis, Department for Children and Adolescents, University Hospital Frankfurt, Goethe University	ralf.schubert@kgu.de
10th author	Melanie	Boerries	German Cancer Consortium (DKTK) and German Cancer Research Center (DKFZ)<br>Institute of Medical Bioinformatics and Systems Medicine, Medical Center-University of Freiburg, Faculty of Medicine, University of Freiburg	melanie.boerries@uniklinik-freiburg.de

Please use an additional sheet if there are more than 10 authors.

SPRINGER NATURE

Change of authorship request form - Journals (pre-acceptance)

Section 3: Please provide a justification for change. Please use this section to explain your reasons for changing the authorship of your manuscript, e.g. what necessitated the change in authorship? Please refer to the (journal) policy pages for more information about authorship. Please explain why omitted authors were not originally included and/or why authors were removed on the submitted manuscript.

Marco Bechtel provided substantial experimental help and expertise with HCoEpiC cells that were used in the revision.

Section 4: Proposed new authorship. Please provide your new authorship list in the order you would like it to appear on the manuscript. Please indicate the corresponding author by adding (CA) behind the name. <u>If the Corresponding Author has changed, please indicate the reason under section 3</u>.

	First name(s)	Family name (this name will appear in full on the final publication and will be searchable in various abstract and indexing databases)	Affiliated institute	E-mail address
1st author	Jindrich	Cinatl Jr.	Institute of Medical Virology, Goethe University Frankfurt	cinatl@em.uni-frankfurt.de
2nd author	Steeve	Boulant	Dept. Mol. Genetics and Microbiology, University of Florda	s.boulant@ufl.edu
3rd author	Sjoerd J.L.	van Wijk (CA)	Institute for Experimental Cancer Research in Pediatrics	s.wijk@kinderkrebsstiftung-frankfurt.de
4th author			Goethe University Frankfurt	
5th author				
6th author				
7th author				
8th author				
9th author				
10th author				

Please use an additional sheet if there are more than 10 authors.

SPRINGER NATURE Springer Nature is one of the world's leading global research, educational and professional publishers, created in May 2015 through the combination of Nature Publishing Group, Palgrave Macmillan, Macmillan Education and Springer Science+Business Media.

Change of authorship request form - Journals (pre-acceptance)

Section 5: Author contribution, Acknowledgement and Disclosures. Please use this section to provide a new disclosure statement and, if appropriate, acknowledge any contributors who have been removed as authors and ensure you state what contribution any new authors made (if applicable per the journal or book (series) policy). Please ensure these are updated in your manuscript - after approval of the change(s) - as our production department will not transfer the information in this form to your manuscript.

New acknowledgements:

New Disclosures (financial and non-financial interests, funding):

S.J.L.v.W. is supported by the Deutsche Forschungsgemeinschaft (DFG) (WI 5171/1-1, FU 436/20-1 and project-ID 259130777 – SFB 1177), the Deutsche Krebshilfe (70113680), the Frankfurter Stiftung für krebskranke Kinder and the Dr. Eberhard and Hilde Rüdiger Foundation. M.B. is supported by the DFG – CRC 850 subprojects C9 and Z1, CRC1479 (Project ID: 441891347- S1), CRC 1160 (Project Z02), CRC1453 (Project ID 431984000 - S1) and TRR167 (Project Z01), the German Federal Ministry of Education and Research by MIRACUM within the Medical Informatics Funding Scheme (FKZ 01ZZ1801B and EkoEstMed–FKZ 01ZZ2015 for G.A.). S.B. was supported by DFG project numbers 415089553 (Heisenberg program), 240245660 (SFB1129), 278001972 (TRR186), and 272983813 (TRR179), the state of Baden-Württemberg (AZ: 33.7533.-6-21/5/1), the BMBF (01KI20198A) and within the Network University Medicine - Organo-Strat COVID-19. M.L.S. was supported by the BMBF (01KI20239B) and DFG project 416072091.

New Author Contributions statement (if applicable per the journal policy):

R.K. performed experiments and analyzed data with help from J.R., S.S. and L.K., M.L.S and S.B. performed SARS-CoV-2 infections and accompanying experiments, gene expression analysis was performed by G.A. and M.B., R.S. provided access and support with the LEGENDplex analysis. M.B., D.B and J.C.Jr. provided the Caco-2 and HCoEpiC cells and expertise. R.K. and S.J.L.v.W. conceived the project and wrote the manuscript. All authors have read, commented and agreed on the submitted version of the manuscript.

State 'Not applicable' if there are no new authors.

SPRINGER NATURE

Change of authorship request form - Journals (pre-acceptance)

Section 6: Declaration of agreement. *All* authors, unchanged, new and removed *must* sign this declaration.
(NB: Please print the form, (docu)-sign and return/upload a scanned copy. Please note that signatures that have been inserted as an image file are acceptable as long as it is handwritten. Typed names in the signature box are unacceptable.) *** Please delete as appropriate. Delete all of the bold if you were on the original authorship list and are remaining as an author.**

	First name	Family name		Signature	Date
1st author	Rebekka	Karlowitz	I agree to the proposed new authorship shown in section 4	R. Karlowitz	13.07.2022
2nd author	Megan L.	Stanifer	I agree to the proposed new authorship shown in section 4		
3rd author	Jens	Roedig	I agree to the proposed new authorship shown in section 4		
4th authors	Geoffroy	Andrieux	I agree to the proposed new authorship shown in section 4		
5th author	Denisa	Bojkova	I agree to the proposed new authorship shown in section 4	[illegible]	13.7.22
6th author	Marco	Bechtel	I agree to the proposed new authorship shown in section 4 **/and the addition of my name to the authorship list**	[illegible]	13.7.22
7th author	Sonja	Smith	I agree to the proposed new authorship shown in section 4	Sonja Smith	14.07.22

SPRINGER NATURE Springer Nature is one of the world's leading global research, educational and professional publishers, created in May 2015 through the combination of Nature Publishing Group, Palgrave Macmillan, Macmillan Education and Springer Science+Business Media.

SPRINGER NATURE

Change of authorship request form - Journals (pre-acceptance)

Section 6: Declaration of agreement. *All* authors, unchanged, new and removed *must* sign this declaration.
(NB: Please print the form, (docu)-sign and return/upload a scanned copy. Please note that signatures that have been inserted as an image file are acceptable as long as it is handwritten. Typed names in the signature box are unacceptable.) *** Please delete as appropriate. Delete all of the bold if you were on the original authorship list and are remaining as an author.**

	First name	Family name		Signature	Date
1st author	Rebekka	Karlowitz	I agree to the proposed new authorship shown in section 4		
2nd author	Megan L.	Stanifer	I agree to the proposed new authorship shown in section 4	[signature]	13-07-2022
3rd author	Jens	Roedig	I agree to the proposed new authorship shown in section 4		
4th authors	Geoffroy	Andrieux	I agree to the proposed new authorship shown in section 4		
5th author	Denisa	Bojkova	I agree to the proposed new authorship shown in section 4		
6th author	Marco	Bechtel	I agree to the proposed new authorship shown in section 4 **/and the addition of my name to the authorship list**		
7th author	Sonja	Smith	I agree to the proposed new authorship shown in section 4		

Change of authorship request form - Journals (pre-acceptance)

Section 6: Declaration of agreement. *All* authors, unchanged, new and removed *must* sign this declaration.
(NB: Please print the form, (docu)-sign and return/upload a scanned copy. Please note that signatures that have been inserted as an image file are acceptable as long as it is handwritten. Typed names in the signature box are unacceptable.) *** Please delete as appropriate. Delete all of the bold if you were on the original authorship list and are remaining as an author.**

	First name	Family name		Signature	Date
1st author	Rebekka	Karlowitz	I agree to the proposed new authorship shown in section 4		
2nd author	Megan L.	Stanifer	I agree to the proposed new authorship shown in section 4		
3rd author	Jens	Roedig	I agree to the proposed new authorship shown in section 4	Jens Roedig	16.07.2022
4th authors	Geoffroy	Andrieux	I agree to the proposed new authorship shown in section 4		
5th author	Denisa	Bojkova	I agree to the proposed new authorship shown in section 4		
6th author	Marco	Bechtel	I agree to the proposed new authorship shown in section 4 **/and the addition of my name to the authorship list**		
7th author	Sonja	Smith	I agree to the proposed new authorship shown in section 4		

SPRINGER NATURE Springer Nature is one of the world's leading global research, educational and professional publishers, created in May 2015 through the combination of Nature Publishing Group, Palgrave Macmillan, Macmillan Education and Springer Science+Business Media.

SPRINGER NATURE

Change of authorship request form - Journals (pre-acceptance)

Section 6: Declaration of agreement. *All* authors, unchanged, new and removed *must* sign this declaration.
(NB: Please print the form, (docu)-sign and return/upload a scanned copy. Please note that signatures that have been inserted as an image file are acceptable as long as it is handwritten. Typed names in the signature box are unacceptable.) *** Please delete as appropriate. Delete all of the bold if you were on the original authorship list and are remaining as an author.**

	First name	Family name		Signature	Date
1st author	Rebekka	Karlowitz	I agree to the proposed new authorship shown in section 4		
2nd author	Megan L.	Stanifer	I agree to the proposed new authorship shown in section 4		
3rd author	Jens	Roedig	I agree to the proposed new authorship shown in section 4		
4th authors	Geoffroy	Andrieux	I agree to the proposed new authorship shown in section 4	[signature]	13.07.2022
5th author	Denisa	Bojkova	I agree to the proposed new authorship shown in section 4		
6th author	Marco	Bechtel	I agree to the proposed new authorship shown in section 4 **/and the addition of my name to the authorship list**		
7th author	Sonja	Smith	I agree to the proposed new authorship shown in section 4		

Change of authorship request form - Journals (pre-acceptance)

	First name	Family name		Signature	Date
8th author	Lisa	Kowald	I agree to the proposed new authorship shown in section 4		15.07.2022
9th author	Ralf	Schubert	I agree to the proposed new authorship shown in section 4		
10th author	Melanie	Boerries	I agree to the proposed new authorship shown in section 4		

Please use an additional sheet if there are more than 10 authors.

In case of author collaborations with formal agreement:

	Name of consortium/consortia	First name	Family name		Signature	Date
Representative/legal guarantor				I agree to the proposed new authorship shown in section 4 **/and the addition/removal*of my name to the authorship list** /and the proposed change in corresponding author		

Both added/removed authors should complete the information in the first table under Section 6.

---- End of form ----

Change of authorship request form - Journals (pre-acceptance)

	First name	Family name		Signature	Date
8th author	Lisa	Kowald	I agree to the proposed new authorship shown in section 4		
9th author	Ralf	Schubert	I agree to the proposed new authorship shown in section 4	R. Schubert	13/07/2022
10th author	Melanie	Boerries	I agree to the proposed new authorship shown in section 4		

Please use an additional sheet if there are more than 10 authors.

In case of author collaborations with formal agreement:

	Name of consortium/consortia	First name	Family name		Signature	Date
Representative/legal guarantor				I agree to the proposed new authorship shown in section 4 **/and the addition/removal*of my name to the authorship list** /and the proposed change in corresponding author		

Both added/removed authors should complete the information in the first table under Section 6.

---- End of form ----

SPRINGER NATURE Springer Nature is one of the world's leading global research, educational and professional publishers, created in May 2015 through the combination of Nature Publishing Group, Palgrave Macmillan, Macmillan Education and Springer Science+Business Media.

SPRINGER NATURE

Change of authorship request form - Journals (pre-acceptance)

	First name	Family name		Signature	Date
8th author	Lisa	Kowald	I agree to the proposed new authorship shown in section 4		
9th author	Ralf	Schubert	I agree to the proposed new authorship shown in section 4		
10th author	Melanie	Boerries	I agree to the proposed new authorship shown in section 4	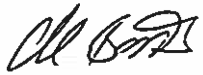	14.07.2022

Please use an additional sheet if there are more than 10 authors.

In case of author collaborations with formal agreement:

	Name of consortium/consortia	First name	Family name		Signature	Date
Representative/legal guarantor				I agree to the proposed new authorship shown in section 4 **/and the addition/removal*of my name to the authorship list** /and the proposed change in corresponding author		

Both added/removed authors should complete the information in the first table under Section 6.

---- End of form ----

SPRINGER NATURE Springer Nature is one of the world's leading global research, educational and professional publishers, created in May 2015 through the combination of Nature Publishing Group, Palgrave Macmillan, Macmillan Education and Springer Science+Business Media.

SPRINGER NATURE

Change of authorship request form - Journals (pre-acceptance)

Section 6: Declaration of agreement. *All* authors, unchanged, new and removed *must* sign this declaration.
(NB: Please print the form, (docu)-sign and return/upload a scanned copy. Please note that signatures that have been inserted as an image file are acceptable as long as it is handwritten. Typed names in the signature box are unacceptable.) *** Please delete as appropriate. Delete all of the bold if you were on the original authorship list and are remaining as an author.**

	First name	Family name		Signature	Date
11st author	Jindrich	Cinatl Jr.	I agree to the proposed new authorship shown in section 4	[signature]	13.07.22
12nd author	Steeve	Boulant	I agree to the proposed new authorship shown in section 4		
13rd author	Sjoerd J.L.	van Wijk	I agree to the proposed new authorship shown in section 4		
4th authors			I agree to the proposed new authorship shown in section 4 **/and the addition/removal*of my name to the authorship list** /and the proposed change in corresponding author		
5th author			I agree to the proposed new authorship shown in section 4 **/and the addition/removal*of my name to the authorship list** /and the proposed change in corresponding author		
6th author			I agree to the proposed new authorship shown in section 4 **/and the addition/removal*of my name to the authorship list** /and the proposed change in corresponding author		
7th author			I agree to the proposed new authorship shown in section 4 **/and the addition/removal*of my name to the authorship list** /and the proposed change in corresponding author		

SPRINGER NATURE

Change of authorship request form - Journals (pre-acceptance)

Section 6: Declaration of agreement. *All* authors, unchanged, new and removed *must* sign this declaration.
(NB: Please print the form, (docu)-sign and return/upload a scanned copy. Please note that signatures that have been inserted as an image file are acceptable as long as it is handwritten. Typed names in the signature box are unacceptable.) *** Please delete as appropriate. Delete all of the bold if you were on the original authorship list and are remaining as an author.**

	First name	Family name		Signature	Date
11st author	Jindrich	Cinatl Jr.	I agree to the proposed new authorship shown in section 4		
12nd author	Steeve	Boulant	I agree to the proposed new authorship shown in section 4	Steeve Boulant	13.08.2022 7
13rd author	Sjoerd J.L.	van Wijk	I agree to the proposed new authorship shown in section 4	[signature]	20.07.2022
4th authors			I agree to the proposed new authorship shown in section 4 /**and the addition/removal*of my name to the authorship list** /and the proposed change in corresponding author		
5th author			I agree to the proposed new authorship shown in section 4 /**and the addition/removal*of my name to the authorship list** /and the proposed change in corresponding author		
6th author			I agree to the proposed new authorship shown in section 4 /**and the addition/removal*of my name to the authorship list** /and the proposed change in corresponding author		
7th author			I agree to the proposed new authorship shown in section 4 /**and the addition/removal*of my name to the authorship list** /and the proposed change in corresponding author		
